# The United Nations convention on the rights of persons with disabilities, neuroscience, and criminal legal capacity

**DOI:** 10.1093/jlb/lsad010

**Published:** 2023-05-17

**Authors:** Benjamin A Barsky, Michael Ashley Stein

**Affiliations:** Edmond & Lily Safra Center for Ethics, Harvard University, Cambridge, MA, USA; Interfaculty Initiative in Health Policy, Harvard University, Cambridge, MA, USA; Harvard Law School, Cambridge, MA, USA

**Keywords:** United Nations Convention on the Rights of Persons with Disabilities, Neuroscience, Criminal Law, Insanity Defense, Human Rights

## Abstract

The United Nations Convention on the Rights of Persons with Disabilities requires states parties to ‘recognize that persons with disabilities enjoy legal capacity on an equal basis with others in all aspects of life.’ This mandate has sparked debate about the interpretation of legal capacity, including within the criminal context as applied to the retrogressively named ‘insanity defense.’ Yet, under-examined are two questions: First, what defenses should defendants with psychosocial disabilities be able to invoke during criminal prosecutions? Second, what kind of evidence is consistent with, on the one hand, determining a defendant’s decision-making capacity to establish culpability and, on the other hand, the right to equal recognition before the law? Developments in neuroscience offer a unique prism to grapple with these issues. We argue that neuroscientific evidence of impaired decision-making, insofar as it presents valid and interpretable diagnostic information, can be a useful tool for influencing judicial decision-making and outcomes in criminal court. In doing so, we oppose the argument espoused by significant members of the global disability rights community that bioscientific evidence of psychosocial disability should be inadmissible to negate criminal responsibility. Such a position risks more defendants being punished harshly, sentenced to death, and placed in solitary confinement.

## I. INTRODUCTION

The United Nations Convention on the Rights of Persons with Disabilities (‘CRPD’)^1^ has created a new paradigm for over one billion people globally by recognizing comprehensive human rights for persons with disabilities.^2^ Nearly universally signed and ratified,^3^ the CRPD requires, among its core provisions, that all states parties ‘recognize that persons with disabilities enjoy legal capacity on an equal basis with others in all aspects of life.’^4^ Much of the literature relating to this directive has focused on the role of supported decision-making in facilitating the expression of an individual’s will and preference, especially in relation to the involuntary receipt of health care.^5^ Less considered yet equally contentious, Article 12’s equal recognition mandate has sparked debate about the interpretation of legal capacity in the criminal context as it applies to the retrogressively named ‘insanity defense.’^6^ Two important and interrelated questions remain under-examined in this respect: First, what defenses, if any, should defendants with psychosocial disabilities^7^ be able to invoke during criminal prosecutions? Second, what evidentiary typology is consistent with, on the one hand, determining a defendant’s decision-making capacity to establish culpability and, on the other, the right to equal recognition before the law?

Developments in neuroscience and law offer a unique and novel prism through which to engage in this two-pronged inquiry. Neuroscience, after all, concerns itself with the brain—the locus of emotion, decision-making, and behavior. By implication, it includes the study of impairments that—together with environmental and other non-biological factors^8^—can manifest disability.^9^ As a result, the role of neuroscience in criminal legal administration raises issues that are important in their own right: Can neuroscientific evidence provide an accurate assessment of decision-making capacity? Is this evidence powerful enough to enable a jury to determine a defendant’s mental state at the time of an offense? These issues are relevant to foundational criminal law concepts—including the nexus of capacity and responsibility—that have yet to be vetted against the CRPD’s disability rights paradigm in general and Article 12’s equal recognition mandate in particular.

This Essay delves into this untouched intersection of law, disability rights, human rights, and bioethics, and asserts that neuroscientific evidence of impaired decision-making, insofar as it presents valid and interpretable diagnostic information, can be a useful tool for influencing judicial decision-making and outcomes in the criminal context. In doing so, we diverge from the argument espoused by significant members of the global disability rights community: that bioscientific evidence of psychosocial disability should not influence the imposition of criminal punishment.^10^ Two premises inform our position. First, neuroscience has evolved—even if slowly and haphazardly—in its ability to identify and diagnose brain and behavioral impairments.^11^ Thus, even if such impairments are often clothed in biomedical terminology, they remain important for individuals with psychosocial disabilities to avoid dignity-depriving prosecution and punishment. Second, neuroscientific evidence of impairments is useful for the crafting and implementation of laws that restrict the imposition of punishment practices causing disparate harm due to those very same people with psychosocial disabilities.^12^

This Essay proceeds in three Parts. Part II discusses the text, history, and ongoing debates about Article 12. It highlights the continuing tension between the core tenet of the predominant ‘absolutist’ notion of legal capacity advanced by the international disability rights community,^13^ and the countervailing assertion by academics, lawyers, and courts that instances exist where formal equality (i.e. treating like as alike) undercuts outcome-based substantive equality (i.e. recognizing and accommodating difference) that is required by the CRPD and disability justice.^14^ Part III focuses on how and when legal actors have used neuroscientific evidence to decide on differential criminal punishment, including sentences, for defendants with psychosocial impairments.

Part IV argues that the absolutist position in general, and its opposition to laws that treat people differently because of evidence of mental disorder in particular, risk creating disproportionate suffering for the very people it seeks to protect. To support this position, we engage three areas of criminal law and administration: (i) sentencing; (ii) the death penalty; and (iii) solitary confinement. Each area reveals that the use of neuroscience has the potential to achieve outcomes that are more just than the absolutist position, while also adhering to Article 12’s call for equal treatment before the law. We conclude with some reflections.

## II. CRIMINAL LEGAL CAPACITY UNDER ARTICLE 12

CRPD Article 12 sets forth the right to equal recognition before the law.^15^ It requires, among other matters, that states ‘recognize that persons with disabilities enjoy legal capacity on an equal basis with others in all aspects of life.’^16^ Beyond this proclamation, the text of Article 12 provides little guidance for applying ‘legal capacity’ in relationship to its demand for equal recognition. As we explain in Part II.A., however, a cursory understanding of the CRPD’s drafting history reveals that this ambiguity was a byproduct of a compromise that occurred during the treaty’s negotiation. Examining that compromise, if only briefly, provides a critical account of how absolutists have come to define legal capacity in the criminal context—an issue that we address in Parts II.B and II.C.

### II.A. Absolutism in the Criminal Context

During the CRPD negotiations, debates about the framing of Article 12 centered on the appropriateness of state-sanctioned substituted decision-making arrangements—commonly known as ‘guardianship’ or ‘conservatorship’—under which a court-appointed individual possesses decision-making over another’s activities.^17^ The question of what to do with substituted decision-making divided negotiators into two camps. The first, generally comprised of state representatives, pushed for language that made explicit room for substituted decision-making.^18^ The second, comprised mainly of representatives of disabled peoples’ organizations (‘DPOs’)—notably the World Network of Users and Survivors of Psychiatry (‘WNUSP’)—argued for the abolition of substituted decision-making. Of relevance, this position reflected opposition among DPOs to laws—including the insanity defense—that denied people with disabilities’ right to make self-determining decisions.^19^

To reach compromise, both groups agreed to leave Article 12 vague about whether to permit or prohibit substituted decision-making. Instead, they converged on language that required states to implement ‘safeguards’ that would ensure that persons with disabilities can express their ‘will and preferences’ without ‘conflict of interest and undue influence.’^20^ Even still, influential interpretations of Article 12 have endorsed and elaborated upon the absolutist stance espoused by many DPOs. According to such interpretations, the provision requires formal equality between all people regardless of disability, treating like as alike. Some of these accounts, moreover, have extended into discussions about criminal law, where conceptualizations of legal capacity bear different significance than under civil law. Below, we synthesize a trilogy of such interpretations, coming from the Office of the United Nations High Commissioner for Human Rights (‘UNHCHR’); the Committee on the Rights of Persons with Disabilities (‘CRPD Committee’); and an *amicus brief* submitted in a case before the International Criminal Court (‘ICC’). We, in turn, distill doctrinal principles that emerge from these mutually reinforcing accounts.

### II.B. Influential Interpretations

#### II.B.1. UNHCHR

Consider, first, the UNHCHR report submitted in January 2009 as part of its effort ‘to enhance awareness and understanding’ of key provisions of the CRPD.^21^ According to the report, Article 12 enshrines the principle that people with disabilities are entitled to the same decision-making ability as everyone else. Under civil law, this principle requires abolition of laws that permit ‘interdiction or declaration of incapacity of persons on the basis of their mental, intellectual or sensory impairment and the attribution to a guardian of the legal capacity to act on their behalf.’^22^ Similarly, under criminal law, it also requires abolition of the insanity defense, which can negate a defendant’s criminal responsibility due to psychosocial disability.^23^ Ostensibly the logic is that psychosocial disability alone cannot rob a person of their volition or decisional power. If it could, laws restricting decision-making ability due to psychosocial disability would continue to be justified, which is exactly the problem that Article 12 seeks to address. The UNHCHR advocates instead for the use of ‘disability-neutral’ doctrine, which would look to ‘the situation of the individual defendant’ to determine whether they had the *mens rea* necessary for criminal liability.^24^

#### II.B.2. CRPD Committee

Next, consider General Comment No. 1 (‘GC1’), which the CRPD Committee promulgated in May 2014 to provide interpretative guidance on Article 12.^25^ GC1 disaggregates the concept of legal capacity into two subcomponents: ‘legal standing’ and ‘legal agency.’^26^ Legal standing refers to recognition of legal personhood, entitling all persons to a comprehensive bundle of public and private rights, including possessing a birth certificate, being a registered voter, having contractual relationships, etc. Legal agency, by contrast, refers to having one’s legal personhood recognized by, and protected under, law. Some examples include having one’s age accounted for when getting certain public benefits; being allowed to vote during elections; and having access to the judicial system to enforce a contract, such as a deed of sale.

GC1 submits that disability status should not usurp legal standing and agency. A psychosocial disability, whatever its impact on a person’s ‘mental capacity’ (i.e. ability to make decisions), cannot serve as a predicate for robbing their freedom to engage in the ever-expanding suite of activities that make life meaningful.^27^ The reason seemingly is that legal standing and agency are independent of someone’s mental capacity. Thus, even if someone’s decision-making abilities are comparatively low, they remain entitled to full legal personhood and protection. This notion supports GC1’s call to abolish ‘substitute decision-making regimes such as guardianship, conservatorship and mental health laws that permit forced treatment.’^28^ Each regime, at least on the CRPD Committee’s interpretation, conditions legal standing and agency on mental capacity. This conception also explains, as we elaborate below, why GC1 proponents disfavor criminal law defenses that discharge defendants of responsibility due to psychosocial disability.

#### II.B.3. ICC Amicus Brief

Finally, consider the *amicus brief* submitted by Tina Minkowitz—WNUSP’s lead representative during the CRPD negotiations—and Robert D. Fleischner (‘Minkowitz & Fleischner brief’) in Dominic Ongwen’s prosecution before the ICC for widespread crimes of war and against humanity.^29^ ICC Trial Chamber IX found Ongwen guilty of 61 such offenses and convicted Ongwen to 25 years of imprisonment.^30^ Ongwen appealed his sentence, arguing, among other things, that the trial chamber failed to account properly for his ‘current mental disabilities’ when deciding his sentence. ^31^This argument prompted the Appeals Chamber to seek guidance on whether the Rome Statute discharges a defendant from criminal responsibility if they suffered from a ‘mental disease or defect’ that destroyed their ‘capacity to appreciate the unlawfulness or nature of [their] conduct, or capacity to control [their] conduct to conform to the requirements of law . . . .’^32^

Enter the Minkowitz & Fleischner brief. Echoing the GC1 and UNHCHR accounts, its core claim is that the Appeals Chamber should reject analysis of ‘mental disease or defect’ altogether.^33^ Instead, the operative legal question should be: ‘Did the defendant’s state of mind at the time of committing the acts amount to *mens rea* . . . taking into account any distress or unusual perceptions he experienced at the time?’^34^ ‘A finding of culpability,’ the brief continues, would not ‘hinge on whether or not a “mental illness” could be deduced in one way or another or on a finding of incapacity that places an individual outside the mutual obligations owed to other members of the community.’^35^ Rather, culpability would turn entirely on evidence relevant to the defendant’s subjective experience at the time of the alleged offense.

### II.C. Emergent Doctrinal Principles

Triangulating the UNHCHR’s account of Article 12, GC1, and the Minkowitz & Fleischner brief brings the absolutist doctrine of criminal legal capacity into focus. First, it requires the abolition of rules like the insanity defense that rely on the diagnosis of an individual’s psychosocial disability to discharge them from criminal responsibility. The logic is that legal recognition of such rules would assume that the diagnosis of having psychosocial disabilities automatically rob individuals of their legal ability to make decisions autonomously. Second, and more controversially, the absolutist doctrine rejects the idea that mental capacity can be impacted by an underlying medical and thus diagnosable condition; in Minkowitz and Fleischner’s words, ‘[m]ental capacity is not an objective, scientific and naturally occurring phenomenon.’^36^ Rather, mental capacity ‘is contingent on social and political contexts, as are the disciplines, professions and practices which play a dominant role in assessing mental capacity.’^37^

As a result of this pair of notions, the absolutist doctrine stands in direct opposition to rules such as that adopted in Norway where defendants are absolved of criminal responsibility if evidence emerges that they suffered from a psychotic disorder at the time of the alleged offense.^38^ More generally, this account, reaffirmed by experts and advocates since the CRPD’s adoption,^39^ reflects resistance to laws and policies that leave room for the differential treatment of persons with psychosocial disabilities. As we argue in Parts III and IV, however, this position gives room to punishment practices that have become the subject of neuroscientific research, and that cause disparate harm to those very same persons with psychosocial disabilities.

## III. NEUROSCIENCE AND CRIMINAL PUNISHMENT

Neuroscientific applications—which have become increasingly common in criminal law, policy, and practice—include neuroimaging technologies like electroencephalography (‘EEG’), computed tomography (‘CAT’) scans, magnetic resonance imaging (‘MRI’), and positron emission tomography (‘PET’) scans.^40^ EEG uses electrodes to measure electrical activity in the brain, allowing for the visual identification of psychotic disorders such as schizophrenia, epilepsy, drug intoxication, and other brain disorders (e.g. Alzheimer’s disease, narcolepsy).^41^ CAT scans use X-ray measurements to create cross-sectional visuals (i.e. slices) of the brain, enabling diagnosticians to detect tumors, lesions, traumatic injuries, and intracranial hemorrhage.^42^ MRI technology leverages computer-generated radio waves and a magnetic field to produce detailed pictures of brain structures, facilitating the diagnosis of strokes, injuries, and psychotic and neurodegenerative disorders.^43^ PET scans employ biochemical compounds (e.g. glucose) that are trackable when metabolized in brain tissue, creating three-dimensional images of functional activity. PET has proven useful for the clinical management of individuals with psychiatric disorder diagnoses, such as mood (e.g. depression) and anxiety disorders, schizophrenia, and obsessive–compulsive disorder.^44^

Lawyers have also used neuroscience at all stages of the criminal adjudication process.^45^ First, litigants can move to contest a defendant’s competence to stand trial during pretrial proceedings, at which point neurobiological evidence can help render more comprehensive competency evaluations. Second, such evidence can emerge at trial to contest *mens rea*.^46^ Third, neurobiological findings, as Nita Farahany has found, now appear ‘clearly entrenched in sentencing decisions.’^47^ Finally, as we elaborate below, these findings have informed the development of laws that bear on punishment practices fallings outside the ambit of judicial decision-making, including with respect to solitary confinement.^48^

Although the most contentious debates surrounding the use of neuroscience in criminal court have gravitated around issues of responsibility (i.e. identifying *mens rea* at the time of an alleged offense)^49^ and deception (i.e. detecting lies),^50^ our assertions are much less controversial. In Part III.A., we address how legal actors have used neuroscience to inform criminal punishment decision-making. In Part III.B., we address the influence of discretion in punishment decision-making, especially as it pertains to the decision maker’s normative assessment and judgment of neuroscientific evidence. This very discretion, we suggest in Part IV, is what raises the importance of analyzing the use of neuroscientific evidence in criminal court through Article 12’s vantage point.

### III.A. Judicial and Nonjudicial Criminal Punishment

We now turn to neuroscience’s influence on criminal punishment—a domain comprising an amalgamation of law (e.g. death penalty); policy (e.g. legislation, administrative decision-making); and politics (e.g. public opinion, tough-on-crime promises). This examination reveals how neuroscientific evidence of psychiatric disorder—which can emerge during pretrial proceedings (e.g. competency-to-stand-trial assessments) or at trial (e.g. insanity defense evaluations and testimony)—bears on judicial and nonjudicial punishment decision-making. It, in turn, establishes the important relationship between the evidence collection process and the criminal legal treatment of defendants with psychosocial disabilities.

Consider first *judicial punishment*. We understand this category to include judge-imposed criminal sanctions, such as incarceration sentences. Generally, judges have used neuroscientific evidence to decrease, or ‘mitigate,’ sentences.^51^ They also have license to increase, or ‘aggravate,’ sentences using the same evidence. The reason for this latitude is that rules of evidence at the sentencing stage are liberal. Under the Federal Sentencing Guidelines, evidence is introducible if it has ‘sufficient indicia of reliability to support its probable accuracy,’^52^ and as Maneka Sinha has found, this standard is similar under both capital and most state cases.^53^ As a result, tools like brain scans ‘may be used in sentencing proceedings to identify and support claims of lesser culpability due to circumstances beyond the control of the offender that could have a mitigating effect on the sentence.’^54^

Calls have also grown to use neuroscientific evidence to avoid imposing the death penalty.^55^ For example, in the widely reported case of Grady Nelson, a jury chose not to impose the death penalty because EEG analyses revealed ‘an obvious abnormality in the left frontal lobe’ that resulted from traumatic brain injuries.^56^ This example was among the first in a wave of cases that relied on neuroimaging data to justify imposing lesser punishment.^57^ Likewise, in the area of juvenile justice, the U.S. Supreme Court has used neuroscientific evidence to find the death penalty, life sentences without parole for nonhomicide offenses, and mandatory life sentences without parole unconstitutional under the Eighth Amendment of the Constitution.^58^

Neuroscientific evidence has also influenced *nonjudicial punishment*, which we mean to include criminal legal practices that generally lie outside the realm of judicial decision-making, such as solitary confinement. This area is vast as it encompasses the multiple domains of criminal administration and practice subject to legislative and administrative decision-making. It is, in this sense, a political domain. For example, researchers in a nationally representative survey study on the public’s approval of neurolaw found that ‘when neurolaw was framed as being helpful to criminal defendants, Republicans were less likely to approve’ than Democrats and Independents.^59^ At the same time, according to Francis Shen, neuroscience continues to have ‘the potential to be transformative’ outside the courtroom.^60^ New York’s Humane Alternatives to Long-Term Solitary Confinement Act (‘HALT Act’)—which bans the use of solitary confinement for people with serious mental illness, among other things—cites ‘the physical and psychological effects of segregated confinement.’^61^ This law is an example among many where legislative and administrative actors can use their policy discretion to bring about change in criminal legal practices.

### III.B. Discretion

How neuroscientific evidence influences criminal punishment is, by and large, a matter of discretion. Just as lawmakers and administrative policy-makers can justify policies based on relevant empirical research, trial judges can identify facts from the evidentiary record—including neuroscientific findings of impairments—to make punishment decisions. For example, without a mandatory sentencing scheme, judges can sentence defendants within broad ranges.^62^ Indeed, in many U.S. states, judges are commonly able to pick a sentence between 0 and 20 years in prison.^63^ That choice—at its core an adjudicative judgment about whether the nature and degree of impairment should affect criminal legal outcomes—necessarily depends on assessment of mitigating or aggravating facts presented during proceedings.

Another important yet often neglected aspect of criminal punishment discretion concerns violations of community supervision. Almost all individuals incarcerated in prisons will re-enter society, but their release often hinges on the fulfillment of specific conditions. Some on probation can complete their sentence by not re-offending and reporting periodically to their probation officers.^64^ Others, at the risk of violating their probation, have to comply with a laundry list of terms that limit their ability to enjoy community activities, force them into programs and treatment, and require financial responsibilities.^65^ If a condition violation occurs, however, a revoking judge detains discretion to impose a sanction, including renewing the probation order or reimposing a term of incarceration. The adjudication of community supervision violations then becomes another point where an individual’s history, as documented by the case’s evidentiary record, can influence punishment decision-making.

## IV. ARTICLE 12 AND NEUROSCIENCE IN THE CRIMINAL CONTEXT

We have so far juxtaposed two descriptive ideas. First, Article 12 defines legal capacity in non-specific terms. It opts instead for a regulatory scheme under which states must enact safeguards that maximize self-determination and decision-making autonomy. At the same time, the international disability rights community and U.S. supporters have interpreted Article 12 in absolutist terms, rejecting measures such as the insanity defense that give differential treatment to persons with and without disabilities. Second, lawyers, judges, and policy-makers have increasingly relied on neuroscientific evidence to assess the cognitive makeup of criminal defendants with psychiatric impairments, and, in turn, to influence judicial and nonjudicial punishment decision-making.

This Part argues against these absolutist interpretations of Article 12. We begin by outlining flaws in the absolutist doctrine. Next, we focus on the doctrine’s peculiar rejection of the idea that psychiatric impairments are not objectively measurable phenomena. We contend that, in doing so, absolutism closes out the possibility of using neurodiagnostic evidence to achieve just outcomes for defendants with psychosocial disabilities. This discussion leads into our concluding thoughts, where we suggest that to align with the CRPD’s purpose and principles, interpretations of Article 12 must seek to account for and achieve outcome-based substantive equality.

### IV.A. Doctrinal Limitations

The absolutist doctrine embeds at least three normative commitments that merit scrutiny. The first is its opposition to laws and policies that use psychiatric diagnoses as touchstones for the differential allocation of rights and benefits. We are concerned about the substantive scope of this opposition, and about the type of evidence that the doctrine would deem admissible versus inadmissible. The doctrine’s proponents have taken a clear position with respect to the insanity defense. Yet they have failed to extend it to other criminal law doctrines, including competency to stand trial and the intoxication defense^66^, and to areas of civil and administrative law where findings of mental illness or other behavioral health conditions are necessary for the receipt of benefits or services.^67^

This concern dovetails with jurisprudential uncertainty about the doctrine’s over-generality. In a watershed opinion, the German Federal Constitutional Court (‘GFCC’) held that the state’s duty to protect warrants forced medical treatment in limited circumstances, including when citizens lack the necessary competence (or ‘free will’) to render informed medical decision-making.^68^ Although seemingly in contravention with GC1, the GFCC found that the CRPD Committee, in fact, never addressed whether medical emergencies justify coercion when patients cannot provide informed consent.^69^ In this way, it read Article 12(4) to allow for the exercise of coercion, so long as safeguards against conflicts of interest and abuse are in place.^70^ As a result, the GFCC’s decision casts into doubt the CRPD Committee’s univocal authoritativeness on interpretational questions surrounding Article 12, and it also spotlights the doctrine’s unresolved boundaries.

Another concern is the related notion that defenses that rely on assessments of mental capacity are stigmatizing because, as Minkowitz and Fleischner suggest, they rob ‘persons with disabilities of equal standing in their communities and reinforce[] practices of exclusion and segregation such as guardianship or involuntary psychiatric treatment.’^71^ The relationship between capacity-based defenses and the sanctioning of liberty-depriving practices is not as clear as they suggest. To the contrary, such defenses can prevent the criminal institutionalization of persons whose minds did not, or were not able to, show the requisite blameworthiness to commit a crime. The question that follows, then, is whether people found not guilty by reason of incapacity are necessarily subject to coercive mechanisms outside the criminal context and permitted under civil law, including those listed by Minkowitz and Fleischner. The answer there will depend on jurisdictional preferences and the availability of non-coercive schemes like supported decision-making arrangements, which an increasing number of countries have adopted in the wake of the CRPD—a development that we wholeheartedly support.

Moreover, Article 12 absolutist theory assumes that protection from criminal liability because of a finding of mental illness relieves certain people from broadly shared social responsibilities. By consequence, the theory goes, two classes of people arise: One that can be punished because of their sound-mindedness, and another that cannot because of their mental illness. We recognize that this classification may cause harm, not least by systematizing the belief that people with mental illness lack the capacity to obey the commands of the law and, as a result, warrant peculiar sociolegal treatment. But this harm vanishes the moment the same defenses become available to those with and without mental illness.^72^ Thus, the absolutist theory is problematic because of its opposition to capacity-based defenses that utilize diagnostic exercises during the criminal adjudicatory process to determine capacity. As long as psychiatric assessment—rather than preexisting diagnosis or labeling—can inform a trier of fact about the presence of *mens rea* or the existence of justification or duress in the individuals before them, it does not *per se* discriminate against defendants with psychosocial disabilities. Rather, it constitutes evidence that allows for the substantiation of defenses available to anyone, regardless of mental illness or disability more generally.

The third is the doctrine’s rejection of mental capacity being ‘an objective, scientific and naturally occurring phenomenon’^73^—a proposition that we interpret as deriving from the doctrine’s concomitant repudiation of the biomedical model of disability. We have already made the case that health care providers are increasingly leaning on neuroscientific tools to assess brain activity, detect abnormalities, and make diagnoses. This trend is also occurring in other fields of bioscientific research, including genetics, where psychiatric disabilities and psychopathology more generally are understood in biological terms rather than as social constructions.^74^ Across these still-developing fields, researchers, and practitioners have started making strides in bridging the chasm between the mapping of biomarkers and clinical decision-making, including through methodological applications like machine learning that can facilitate the translation of brain activity into clinical outcomes.^75^

### IV.B. Doctrinal Implications

By dismissing outright science’s ability to achieve this translational task, absolutism risks setting a stubborn precedent by which people with psychosocial disabilities are deprived of potential accommodative benefits. In the penal context, this posture also runs countercurrent to jurisprudential and legislative developments where mental illness is considered to be a sufficient basis for the availability of merciful and rehabilitative practices. We alluded above to three domains where such is the case: (i) sentencing reform; (ii) the death penalty; (iii) and solitary confinement. We expand on this discussion below and contend that, across each of these domains, neurodiagnostic tools and assessments have the potential to help achieve more just outcomes for defendants with psychosocial disabilities.

#### IV.B.1. Sentencing

We have already noted that defendants routinely invoke neuroscientific evidence to mitigate criminal legal punishment. With increasing sophistication of neurodiagnostic tools, this trend will likely continue unabated.^76^ Furthermore, neuroscientific evidence can be harnessed for purposes other than reducing the length and intensity of punishment, ^77^ including, as Francis Shen has proposed, ‘to individualize criminal sentences and tailor probation and parole to meet the unique profile of each offender.’^78^ The advent of computational and precision psychiatry supports this effect. This still-developing line of work seeks to enable mental health diagnoses and treatments based on multidimensional information such as the environment in which people live as well as their biological makeup.^79^ Such an approach, as described by its proponents, ‘resolves the dialectic’ between the often-problematic diagnostic categories offered by the Diagnostic and Statistical Manual of Mental Disorders and latent constructs of psychopathology, such as those identified by the Research Domain Criteria project.^80^

These developments are positive as far as they achieve a degree of reform, but they bear the high and heavy risk of not going far enough.^81^ Changes in sentencing practices, to state the obvious, do not go to the roots of mass criminalization of communities of color, poverty, and disability. For people with compromised decision-making in particular, mere detention or incarceration can trigger a potentially devastating and irreversible cascade effect.^82^ The question that arises, then, is whether neuroscience-informed sentencing reform will constitute one step toward halting the blunt and indiscriminate use of criminal legal sanctions against historically marginalized populations. On this question, we remain skeptical.

#### IV.B.2. Death Penalty

We observed above that the Supreme Court relied on behavioral scientific findings—particularly the notion that youth lack maturity and a fully developed sense of responsibility—to ban the death penalty for juveniles.^83^ That decision built on the 2002 ruling in *Atkins v. Virginia* holding that execution of people with intellectual disabilities violates the U.S. Constitution’s prohibition against cruel and unusual punishment—a holding that, according to Justice John Paul Stevens, follows from how ‘our society views mentally retarded offenders as categorically less culpable than the average criminal.’^84^ Although we resist the Court’s distasteful denomination of people with intellectual disabilities, Justice Stevens’s observation that Americans generally oppose state-sanctioned execution of people with psychosocial remains, by and large, correct.^85^

The United States is once again not alone in limiting the death penalty’s reach to people with compromised decision-making. For example, the Council of the European Union has conditioned membership into the European Union in part on prohibition of the death penalty for ‘[p]ersons suffering from any mental illness or having an intellectual disability.’^86^ Other countries, including India, Japan, and Pakistan, also have measures limiting application of the death penalty to people with mental illness.^87^ This pattern, consistent with the customary international law principle forbidding application of the ‘death sentence . . . on persons who have become insane,’^88^ suggests recognition of the cruelty of carrying out executions without due regard to moral considerations.^89^

#### IV.B.3 Segregation Practices

The human rights community has condemned solitary confinement and other segregation practices given their associated physical and mental harms, especially as experienced by people with psychosocial disabilities. This advocacy movement has fueled the development of laws and policies banning or limiting segregation practices for people with psychiatric disabilities. For example, as we noted in Part III, New York State’s HALT Act prohibits ‘segregated confinement’ for incarcerated people with serious mental illness.^90^ Connecticut’s General Assembly also passed a law limiting the use of ‘isolated confinement’ for members ‘of a vulnerable population,’ including people with mental or developmental disabilities, medically vulnerable individuals, and those with histories of psychiatric hospitalization or self-harm.^91^ Similar laws exist in other countries, including Australia, Bangladesh, Canada, India, Serbia, and Ukraine, where limits exist on the use of solitary confinement for persons with psychosocial disabilities.^92^ They also align with Rule 45(2) of the United Nations Standard Minimum Rules for the Treatment of Prisoners, otherwise known as the Nelson Mandela Rules, which states that ‘solitary confinement should be prohibited in the case of prisoners with mental or physical disabilities when their conditions would be exacerbated by such measures.’^93^

At the same time, because these legal developments remain nascent, segregation practices continue unabated across much of the world. In the United States, 20 per cent of people in prisons and 18 per cent of those in jails had experienced disciplinary segregation, administrative segregation, or solitary confinement in the past year.^94^ For more recent evidence, consider the U.S. Department of Justice’s (‘DOJ’) recent investigation of segregation practices in Massachusetts prisons.^95^ The DOJ found that the Massachusetts Department of Correction’s treatment of those on ‘mental health watch’—a practice reserved for individuals who are at risk of self-injury or suicide—violated the Eighth Amendment on two grounds.^96^ First, the investigation revealed that those on mental health watch suffer from near-total neglect from staff and have regular access to instruments that allow for self-harm and mutilation. Second, people could stay on mental health watch for weeks, even months. During these periods, they are in their cells for at least 23 hours per day without meaningful mental health care, entertainment, or human contact. Similar practices have also persisted in countries across the world, where solitary confinement is common as a means of carceral discipline.

Neuroscience can bear on the legitimacy and legality of carceral segregation practices, not least through understanding of the psychophysiological effects of prolonged isolation and disconnection from social stimuli. Although human research exploring the neurocognitive effects of solitary confinement is underdeveloped—a problem not least due to the obvious ethical implications of such research—animal research ‘strongly supports the notion of altered neuroplasticity as a result of an impoverished environment.’^97^ Consequences include ‘hyperactivity, ambivalence to novelty, altered responses to stressors, cognitive impairments, increased aggression, and alterations in mesolimbic dopamine functioning.’^98^ Other research has found solitary confinement to lead to disruptions of daily routines, undiagnosed and entreated ailments, malnutrition, musculoskeletal pain increases, and cardiovascular and psychological problems.^99^

## V. CONCLUSION

The increased use of neuroscience in the criminal context offers a unique lens through which to assess, and critique, the implications of the absolutist interpretation of legal capacity under CRPD Article 12. Absolutism opposes doctrines like the insanity defense because they rely on psychiatric diagnoses to absolve defendants from criminal responsibility. In doing so, the position goes, such doctrines discharge people with psychosocial disabilities from mutual social responsibility, which is stigmatizing and inconsistent with the CRPD’s mandate of ensuring equal recognition under the law for all. Absolutism also repudiates the idea that psychosocial disabilities are observable and scientifically measurable phenomena.

We assert that absolutism suffers from three notable flaws. First, by rejecting inquiry into defendants’ psychiatric status for criminal defenses, it draws a line that logically extends into other domains of law where the allocation of disability-sensitive and accommodative benefits depends on similar psychiatric evidence. Second, its call to abolish the insanity defense relies on a theory of discrimination that, in fact, is capacious enough to conceptualize disability in biomedical and social terms. And finally, and more notably, it dismisses neuroscientific and other bioscientific insights into the causes and determinants of psychosocial states that are routinely criminalized, and into the consequences of criminal legal practices that are overwhelmingly opposed by the human and civil rights community. These flaws hold special weight in the areas of sentencing, death penalty jurisprudence, and solitary confinement—where a blunt application of absolutist principles would lead to disproportionate suffering for those with psychosocial disabilities.

We assert that a better understanding of criminal legal capacity under Article 12 should make room for the differential treatment of people with disabilities. By accommodating disability through more humane and non-punitive measures, this understanding accounts for the well-documented oppressive and dangerous conditions of carceral systems in the United States and much of the world. It also aligns with the spirit and thrust of the CRPD more generally, which envisions a world where—absent universal design structures—people with disabilities receive accommodations to thrive in society without fear of oppression and domination.

## Footnotes

1 Convention on the Rights of Persons with Disabilities, May 3, 2008, 2515 U.N.T.S. 3 [hereinafter CRPD]. Although the United States has not ratified the CRPD, cities, counties, and states across the country have incorporated Article 12’s principles in laws and policies. *See* Benjamin A. Barsky, *Dual Federalism, Constitutional Openings, and the Convention on the Rights of Persons with Disabilities*, 24 U. Penn. J. Const. L. 345, 365–410 (2022) (providing examples of more than a dozen supported decision-making statutes derived from Article 12).

2
*See generally* Michael Ashley Stein, *Disability Human Rights*, 95 Calif. L. Rev. 75 (2007).

3 OHCHR, *Ratification Status for CRPD—Convention on the Rights of Persons with Disabilities*, United Nations Human Rights Treaty Bodies, https://tbinternet.ohchr.org/_layouts/15/TreatyBodyExternal/Treaty.aspx?Treaty=CRPD&Lang=en (listing the 185 of 193 U.N. member states that have ratified or acceded to the CRPD).

4 CRPD, *supra* note 1, at art. 12; *see generally*  The UN Convention on the Rights of Persons With Disabilities: A Commentary (Ilias Bantekas, Michael Ashley Stein, & Dimitris Anastasiou eds., 2018).

5
*See generally*  Mental Health, Legal Capacity, & Human Rights (Michael Ashley Stein et al. eds., 2021); *see also* text associated with notes 8–9.

6 Notable exceptions include Peter Bartlett, *The United Nations Convention on the Rights of Persons with Disabilities and Mental Health Law*, 75 Modern L. Rev. 752 (2012); Christopher Slobogin, *Eliminating Mental Disability as a Legal Criterion in Deprivation of Liberty Cases: The Impact of the Convention on the Rights of Persons with Disabilities on the Insanity Defense, Civil Commitment, and Competency Law*, 40 Int’l J. L. & Psych. 36 (2015); Jillian Craigie, *Against A Singular Understanding of Legal Capacity: Criminal Responsibility and the Convention on the Rights of Persons with Disabilities*, 40 Int’l J. L. & Psych. 6 (2015); Michael L. Perlin, *God Said to Abraham/Kill Me a Son: Why the Insanity Defense and the Incompetency Status Are Compatible with and Required by the Convention on the Rights of Persons with Disabilities and Basic Principles of Therapeutic Jurisprudence*, 54 Am. Crim. L. Rev. 477 (2017).

7 For purposes of this Essay, we understand psychosocial disabilities to encompass long-term actual or perceived mental, intellectual, developmental, or sensory impairments. *See* CRPD, *supra* note 1, at art. 1; *see also* text associated with notes 74–6.

8 As Emily Murphy explains, disabilities that influence cognitive function do not arise from ‘a simple binary “either/or” of environment or heritable factors. The best available theories explaining the behavioral data are interactionist.’ Emily R.D. Murphy, *Brains Without Money: Poverty as Disabling*, 54 Conn. L. Rev. 699, 718 (2022); *see also generally* Jennifer Karas Montez, Mark D. Hayward, & Douglas A. Wolf, *Do U.S. States’ Socioeconomic and Policy Contexts Shape Adult Disability?*, 178 Soc. Sci. & Med. 115 (2017).

9
*See, e.g.* Niklas Altermark, *The Ideology of Neuroscience and Intellectual Disability: Reconstituting the ‘Disordered’ Brain*, 29 Disability & Soc. 1460 (2014); Stacey A. Tovino, *Will Neuroscience Redefine Mental Injury: Disability Benefit Law, Mental Health Parity Law, and Disability Discrimination Law*, 12 Ind. Health L. REV. 695 (2015).

10 As we explain below, we agree with the notion that a reduction of culpability should not be premised on diagnosis or disability status but instead on a functional determination. And we certainly are in favor of the doctrine being renamed so as not to reinstantiate prejudices against persons with psychosocial disabilities. Rather, our disagreement with the absolutist doctrine centers on the type of evidence that should be allowed during criminal proceedings to substantiate a defendant’s psychosocial disability. *See* infra Part IV.A.

11
*See infra* Part III. For a comprehensive treatment of this issue, *see also*  Dennis S. Charney, Eric J. Nestler, Pamela Sklar, & Joseph D. Buxbaum, Neurobiology Of Mental Illness (5th ed. 2017).

12
*See infra* Part IV.B.

13
*See* Gerald L. Neuman, *Divergent Human Rights Approaches to Capacity and Consent*, *in*  Mental Health, Legal Capacity, & Human Rights 56, 56, et. seq. (Michael Ashley Stein et al. eds., 2021) (coining the term, and explicating divergent approaches to the same challenges).

14
*Compare* Anita Silvers, *Formal Justice, in*  Disability, Difference, Discrimination: Perspectives On Justice In Bioethics And Public Policy 15 (Anita Silvers et al. eds., 1998), *with* Frédéric Mégret, *The Disabilities Convention: Towards a Holistic Concept of Rights*, 12 Int’l J. Hum. Rts. 261 (2008).

15
*See id.* at art. 12.

16
*Id.* at art. 12(1).

17
*See* Barsky, *supra* note 1, at 383–88 (2022) (detailing history of Article 12 negotiations).

18
*See* Barsky, *supra* note 1, at 384 (‘[I]n an early foundational draft crafted in 2003 during a regional workshop in Bangkok, guardianship was the chosen model for regulating the administration of property.’).

19
*See* Faraaz Mahomed, Michael Ashley Stein & Vikram Patel, *Involuntary Mental Health Treatment in the era of the United Nations Convention on the Rights of Persons with Disabilities*, 15 PLoS Med. e1002679 (2018) (identifying the WNUSP’s advocacy as a source of Article 12’s content); *see also* WNUSP, *Implementation Manual for the United Nations Convention on the Rights of Persons with Disabilities* (Feb. 2008), http://wnusp.rafus.dk/documents/WNUSP_CRPD_Manual.pdf (suggesting that WNUSP’s interpretation of the CRPD will result in ‘the abolition of mental health commitment laws, guardianship, and the insanity defense’).

20 CRPD, *supra* note 1, at art. 12(4).

21 UNHCHR, *Thematic Study on enhancing awareness and understanding of the Convention on the Rights of Persons with Disabilities*, ¶ 2, U.N. Doc. A/HRC/10/48 (Jan. 26, 2009).

22
*Id.* at ¶ 45.

23
*Id.* at ¶ 47.

24
*Id.*

25 Comm. on the Rts. of Persons with Disabilities, Gen. Comment No. 1, U.N. Doc. CRPD/C/GC/1 (May 19, 2014) [hereinafter GC1].

26
*Id.* at ¶ 13.

27
*Id.*

28
*Id.* at ¶ 7.

29 The Prosecutor v. Dominic Ongwen, ICC-02/04–01/15A, Appeals Chamber, Amicus Curiae Observations Regarding the Relevance to this Case of the Convention on the Rights of Persons with Disabilities (Dec. 21, 2021) [hereinafter Minkowitz & Fleischner Brief].

30 The Prosecutor v. Dominic Ongwen, ICC-02/04–01/15, Trial Chamber IX, Trial Judgment (Feb. 4, 2021).

31 The Prosecutor v. Dominic Ongwen, ICC-02/04–01/15, Appeals Chamber, Defense Notice of Appeal of the Sentencing Decision (June 28, 2021).

32 Rome Statute of the International Criminal Court, July 17, 1998, art. 31(1)(a), UN Doc. A/CONF.183/9* [hereinafter ICC Statute].

33 To substantiate its position, the brief cites and claims to be ‘consistent’ with a much-cited article by Christopher Slobogin, which argues for the abolition of the insanity defense in favor of a doctrine whereby defendants ‘have a complete defensive claim only when they lack mens rea or act for reasons that sound in justification or duress.’ *See* Minkowitz & Fleischner Brief, *supra* note 29, at ¶ 40 (citing Christopher Slobogin, *An End to Insanity: Recasting the Role of Mental Illness in Criminal Cases*, 86 Va. L. Rev. 1199 (2000)). Yet, although the Minkowitz and Fleischner brief reach the same conclusion as Slobogin—that the insanity defense should be replaced by another, less targeted doctrine—Slobogin does not reject inquiry into a defendant’s mental status when determining whether they lacked *mens rea* or acted in justification or duress. *Id.* at 1207–8. We recognize that the language under Article 30 is similar to the so-called insanity defense under the American Law Institute’s Model Penal Code (MPC), which is the locus of much of Slobogin’s critique. *See* Model Penal Code, § 4.01 (P.O.D. 1962). But whether, in practice, application of Article 30 has given rise to results similar to those in states that have carried over the MPC’s doctrine in their criminal codes remains an open question.

34 Minkowitz & Fleischner Brief, *supra* note 29, at ¶ 8. Article 30 limits criminal responsibility to those who have committed criminal acts ‘with intent and knowledge.’ *See* ICC Statute, *supra* note 32, at art. 30

35 Minkowitz & Fleischner Brief, *supra* note 29, at ¶ 8.

36
*Id.* at ¶ 27. Note that this language, which is not in quotations in the Minkowitz and Fleischner brief, appears verbatim in GC1. *See* GC1 at ¶ 14 (‘Mental capacity is not, as is commonly presented, an objective, scientific and naturally occurring phenomenon.’) Furthermore, given the broad—and sweeping—nature of this language, we interpret it as swallowing instances where defendants might use bioscientific evidence in general and neuroscientific evidence in particular merely to corroborate an argument that their subjective experiences amounted to lacking the necessary *mens rea* at the time of their alleged offense.

37
*Id.*

38
*See* Michael S. Moore, *The Quest for a Responsible Responsibility Test: Norwegian Insanity Law After Breivik*, 9 Crim. L. & Phil. 645, 647 (2015) (‘A defendant [in Norway] who otherwise meets the requirements for criminal conviction (act, intent, causation, etc.) is nonetheless not punishably guilty if at the time he did the criminal act he was “psychotic.”’).

39
*See e.g.* Tina Minkowitz, *Reparation for Psychiatric Violence: A Call to Justice, in*  Mental Health, Legal Capacity, & Human Rights 44 (Michael Ashley Stein et al. eds., 2021) (‘States must abolish and prohibit all regimes of substituted decision-making.’).

40
*See* Francis X. Shen, *Neuroscience, Mental Privacy, and the Law*, 36 Harv. J. L. & Pub. Pol’y 653, 658 (2013); Nita A. Farahany, *Neuroscience and Behavioral Genetics in US Criminal Law: An Empirical Analysis*, 2 J. L. Biosciences 485, 490 (2016).

41
*See e.g.* C. D. Binnie & P. F. Prior, *Electroencephalography*, 57 J. Neurology, Neurosurgery & Psychiatry 1308 (1994); Shelagh J.M. Smith, *EEG in the Diagnosis, Classification, and Management of Patients with Epilepsy*, 76 J. Neurology, Neurosurgery & Psych. ii2 (2005); Muhammad A. Parvaz et al., *Neuroimaging for Drug Addiction and Related Behaviors*, 22 Rev. Neurosci. 609 (2011); Janir Ramos da Cruz et al., *EEG Microstates Are a Candidate Endophenotype for Schizophrenia*, 11 Nature Comm. 1 (2020).

42
*See e.g.* Elizabeth George, et al., *Introduction to Neuroimaging*, 131 Am. J. Med. 346 (2018).

43
*See e.g.* Neurological Diagnostic Tests and Procedures Fact Sheet, Nat. Inst. Neurological Dis. STROKE, https://www.ninds.nih.gov/neurological-diagnostic-tests-and-procedures-fact-sheet; Philip Scheltens et al., *Structural Magnetic Resonance Imaging in the Practical Assessment of Dementia: Beyond Exclusion*, 1 Lancet Neurology 13 (2002); J. M. U-King-Im et al., *Utility of an Ultrafast Magnetic Resonance Imaging Protocol in Recent and Semi-Recent Strokes*, 76 J. Neurology, Neurosurgery & Psych. 1002 (2005); Theodore D. Satterthwaite & Justin T. Baker, *How Can Studies of Resting-State Functional Connectivity Help Us Understand Psychosis as a Disorder of Brain Development?*, 30 Curr. Op. Neurobio. 85 (2015).

44
*See e.g.* M. S. George, T. A. Ketter & R. M. Post, *SPECT and PET Imaging in Mood Disorders*, 54 J. Clinical Psych. 6 (1993); Janine Doorduin et al., *Neuroinflammation in Schizophrenia-Related Psychosis: A PET Study*, 50 J. Nuclear Med. 1801 (2009); Simon Cervenka et al., *Application of Positron Emission Tomography in Psychiatry—Methodological Developments and Future Directions*, 12 Trans. Psych. 1 (2022).

45
*See* Farahany, *supra* note 40, at 488 (‘Criminal defendants regularly use neuroscience at every stage of the criminal process, from pretrial, to trial, and sentencing determinations.’).

46 That said, legal and scientific consensus suggests that neuroimaging tools such as brain scans are unreliable and therefore of little use in determining past mental states. *See* Owen D. Jones & Francis X. Shen, *Law and Neuroscience in the United States*, *in*  International Neurolaw: A Comparative Analysis 349 (2012) (Tade Matthias Spranger ed., 2012) (‘Because the most legally relevant thoughts are likely to be those that occurred in the past. .. brain scans taking place long after the behavior may be of limited diagnostic or forensic use.’); *see generally* Teneille Brown & Emily R.D. Murphy, *Through a Scanner Darkly: Functional Neuroimaging as Evidence of a Criminal Defendant’s Past Mental States*, 62 Stan. L. Rev. 1119 (2009).

47 Farahany, *supra* note 40, at 504.

48 See e.g. Dana G. Smith, *Neuroscientists Make a Case against Solitary Confinement*, Scientific American, https://www.scientificamerican.com/article/neuroscientists-make-a-case-against-solitary-confinement/; Jules Lobel & Huda Akil, *Law & Neuroscience: The Case of Solitary Confinement*, 147 Daedalus 61 (2018).

49
*See e.g.* Nicholas J. Schweitzer et al., *Neuroimages as Evidence in a Mens Rea Defense: No Impact.*, 17 Psych., Pub. Pol. & L. 357 (2011); Uri Maoz & Gideon Yaffe, *What Does Recent Neuroscience Tell us about Criminal Responsibility?*, 3 J. L. & Biosciences 120 (2016); Christopher Slobogin, *Neuroscience Nuance: Dissecting the Relevance of Neuroscience in Adjudicating Criminal Culpability*, 4 J. L. & Biosciences 577 (2017).

50
*See e.g.* Frederick Schauer, *Can Bad Science be Good Evidence-Neuroscience, Lie Detection, and Beyond*, 95 Cornell L. Rev. 1191 (2009); Paul S. Appelbaum, *Law & Psychiatry: The New Lie Detectors: Neuroscience, Deception, and the Courts*, 58 Psych. Serv. (2007); Elena Rusconi & Timothy Mitchener-Nissen, *Prospects of Functional Magnetic Resonance Imaging as Lie Detector*, 7 Frontiers In Hum. Neuroscience 594 (2013).

51 Shelley Batts, *Brain Lesions and their Implications in Criminal Responsibility*, 27 Behavioral Sci. & L. 261, 263 (2009) (‘Neuroimaging has also been presented as evidence for mental illness and sentencing mitigation,. .. although the presentation of neurological evidence as part of a criminal defense or sentencing mitigation is more common.’)

52 U.S.S.G. 6A1.3(a).

53
*See* Maneka Sinha, *Junk Science at Sentencing*, 89 Geo. Wash. L. Rev. 52, 87–88 (2021). Although the bar for introducing neuroscientific evidence is lower in capital than non-capital cases, an increasing number of non-capital cases have featured evidentiary tools like medical records and neuropsychological tests. *See* Brown & Murphy, *supra* note 46, at 1133–34; Farahany, *supra* note 40, at 494.

54 Elizabeth O. Bennet, *Neuroscience and Criminal Law: Have We Been Getting It Wrong for Centuries and Where Do We Go from Here?*, 85 Fordham L. Rev. 437, 449.

55
*See* O. Carter Snead, *Neuroimaging and the ‘Complexity’ of Capital Punishment*, 82 N.Y.U. L. Rev. 1265, 1300 (‘Reported cases and public commentary demonstrate that cognitive neuroscientists are increasingly contributing to the mitigation efforts of capital defendants.’) (internal citations omitted).

56 Greg Miller, *Brain Exam May Have Swayed Jury in Sentencing Convicted Murderer*, 14 Science (2010).

57
*See* Farahany, *supra* note 40, at 504.

58
*See Roper v. Simmons*, 543 U.S. 551 (2005); *Graham v. Florida*, 560 U.S. 48 (2010); *Miller v. Alabama*, 564 U.S. 460 (2012).

59 Francis X. Shen & Dena M. Gromet, *Red States, Blue States, and Brain States: Issue Framing, Partisanship, and the Future of Neurolaw in the United States*, 658 AAAPSS 86, 95 (2015).

60 Francis X. Shen, *Neurolegislation: How U.S. Legislators Are Using Brain Science*, 29 Harv. J. L. & Tech. 495, 519 (2016).

61 2021 NY A.B. 2277 (NS).

62 Rachel Elise Barkow, Prisoners Of Politics: Breaking The Cycle Of Mass Incarceration 196 (2019).

63
*Id.*

64
*See* Anat Kimchi, *Investigating the Assignment of Probation Conditions: Heterogeneity and the Role of Race and Ethnicity*, 35 J. Quant. Crim. 715, 716 (2019) (reviewing the literature on conditions of probation).

65
*Id.*

66 Whether absolutists would oppose the intoxication defense is a compelling question on its own, given the opaque, and often fraught, boundary between disability and drug use. *See e.g.* Leslie Francis, *Illegal Substance Abuse and Protection from Discrimination in Housing and Employment: Reversing the Exclusion of Illegal Substance Abuse as a Disability*, 2019 Utah L. Rev. 902 (2019).

67 As an obvious example, in many if not most developed countries, welfare benefits such as disability insurance are available to people with documented behavioral health conditions. *See* Robert E. Drake et al., *Mental Health Disability: An International Perspective*, 23 J. Disability Pol. Stud. 110, 111–12 (2012) (reviewing international trends). Absolutists have yet to articulate whether such policies are similarly problematic, and if not, why.

68
*See generally* Order of the First Senate of July 26, 2016, 1 BvL 8/15, German Federal Constitutional Ct. BVerfG), ¶ 4, English translation available at http://www.bverfg.de/e/ls20160726_1bvl000815en.html.

69
*Id.* at ¶ 91.

70
*Id.* at ¶ 88.

71 Minkowitz & Fleischner Brief, *supra* note 29, at ¶ 44.

72
*See* Slobogin, *supra* note 33, at 1222 (‘Current insanity tests are overbroad because, if taken literally, they move too far toward the deterministic reductio ad absurdum that no one is responsible. ... Allowing subjectively defined defensive doctrines to do the work better captures the universe of people who should be excused.’).

73 Minkowitz & Fleischner Brief, *supra* note 29, at ¶ 27.

74
*See, e.g*. Jon McClellan & Mary-Claire King, *Genomic Analysis of Mental Illness: A Changing Landscape*, 303 JAMA 2523 (2010).

75
*See, e.g.* Choong-Wan Woo et al., *Building Better Biomarkers: Brain Models in Translational Neuroimaging*, 20 Nature Neurosci. 365 (2017).

76 To this effect, a study of ˃1585 judicial opinions spanning from 2005 to 2012, found that neuroscientific and behavioral genetic evidence has increasingly justified mitigated sentences. *See* Farahany, *supra* note 40, at 504 (finding that, of all opinions analyzed, ‘[a]pproximately 44 per cent of the neurobiological claims raised were attempts to mitigate sentencing.’).

77 See Francis X. Shen, *Computational Psychiatry for Precision Sentencing in Criminal Law*, Bill Of Health (Feb. 17, 2021), http://blog.petrieflom.law.harvard.edu/2021/02/17/computational-psychiatry-precision-sentencing/.

78
*Id.*

79
*See generally* Brisa S. Fernandes et al., *The New Field of ‘Precision Psychiatry,’* 15 BMC Medicine 1 (2017).

80 Karl J. Friston, A. David Redish & Joshua A. Gordon, *Computational Nosology and Precision Psychiatry*, 1 Comp. Psych. 2 (2017).

81 As we observe later in this section, we are more supportive of using neuroscientific evidence for purposes of deflecting people with psychosocial disabilities away from the criminal legal system and achieving decarceration outcomes. *See infra* notes 102–112 and accompanying text.

82
*See* Prison Policy Initiative, *Research Roundup: Incarceration Can Cause Lasting Damage to Mental Health* (May 13, 2021), https://www.prisonpolicy.org/blog/2021/05/13/mentalhealthimpacts/ (collecting sources).

83 Cf. Peter A. Alces, The Moral Conflict Of Law And Neuroscience 60 (2018) (‘Recall *Roper v. Simmons. ...* Writing for the majority, Justice Kennedy took account of neuroscientific insights into adolescent brain development. The Court did not consider the state of Simmons’s brain in particular, but instead relied on studies of neural development generally.’).

84 536 U.S. 304, 316 (2002).

85 Rebecca Beitsch, *States Consider Barring Death Penalty for Severely Mentally Ill*, The Pew Charitable Trusts (2017), http://pew.org/2pBsC3I.http://pew.org/2pBsC3I.

86 U.N. Secretary-General, *Capital Punishment and Implementation of the Safeguards Guaranteeing Protection of the Rights of Those Facing the Death Penalty*, ¶ 90, U.N. Doc. E/2010/10 (Dec. 18, 2009).

87
*See generally* Dhananjay Mahapatra, *India Joins Nations which Don’t Execute Mentally Ill Convicts*, The Times Of India, June 2, 2019, https://timesofindia.indiatimes.com/india/india-joins-nations-which-dont-execute-mentally-ill-convicts/articleshow/69615922.cms;https://timesofindia.indiatimes.com/india/india-joins-nations-which-dont-execute-mentally-ill-convicts/articleshow/69615922.cms; Hiroko Kashiwagi & Naotsugu Hirabayashi, *Death Penalty and Psychiatric Evaluation in Japan*, 9 Frontiers In Psych. 550 (2018); Asad Hashim, *Pakistan’s Top Court bans Execution of People with Mental Illness*, Al Jazeera (2021), https://www.aljazeera.com/news/2021/2/11/pakistani-apex-court-bans-execution-of-mentally-ill-patients.https://www.aljazeera.com/news/2021/2/11/pakistani-apex-court-bans-execution-of-mentally-ill-patients.

88 Comm’n on H.R., Econ. & Soc. Council, Civil and Political Rights, Including Questions of Disappearances and Summary Executions, ¶ 69, UN Doc. E/CN.4/2000/2 (Jan. 25, 2000).

89 We have avoided defining what psychosocial disabilities should trigger a prohibition on the death penalty, in part because that inherently political exercise would be beyond the scope of this Essay. Rather, our focus is on delineating how jurisdictions across the world have limited the death penalty’s application to this population.

90 2021 NY A.B. 2277 (NS), *supra* note 61.

91 Connecticut S.B. 1059, LegiScan (2021), https://legiscan.com/CT/text/SB01059/id/2418145; *see also* Kelan Lyons, *CT Prison System Starts Revising Solitary Confinement Policies*, Ct Mirror (2021), http://ctmirror.org/2021/10/26/spurred-by-executive-order-prison-system-starts-revising-solitary-confinement-policies/. Governor Ed Lamont vetoed the law in June 2021, explaining that it ‘places unreasonable and dangerous limits on the use of restraints.’ Christopher Keating, *Bill Limiting Solitary Confinement goes to Connecticut Gov. Ned Lamont for signature—Hartford Courant*, Hartford Courant (Apr. 29, 2022), https://www.courant.com/politics/hc-pol-solitary-confinement-headed-lamont-20220429-y6a2yraoujhu7g6gk37jctdsla-story.html.

92 Weil, Gotshal & Manges LLP, Cyrus R. Vance Center for International Justice, and Center for Human Rights & Humanitarian Law, American University Washington College of Law, Seeing Into Solitary II: A Further Review Of Solitary Confinement in Certain Nations 41–2 (2021).

93 United Nations Standard Minimum Rules for the Treatment of Prisoners (the Nelson Mandela Rules), G.A. Res. 70/175 (Dec. 17, 2015), Rule 45(2).

94
*See* Allen J. Beck, *Use of Restrictive Housing in U.S. Prisons and Jails, 2011–12*, Bureau Just. Stat. 1, 1 (2015).

95
*See generally* Justice Department Alleges Conditions at Massachusetts Department of Correction Violate the Constitution, U.S. Department Of Justice (Nov. 17, 2020), https://www.justice.gov/usao-ma/pr/justice-department-alleges-conditions-massachusetts-department-correction-violate; Benjamin A Barsky, *Ending Restrictive Housing in Prisons for People with Mental Disorders*, Psych. Serv. (2022).

96
*See generally* U.S. Department Of Justice, *supra* note 95.

97 Lobel & Akil, *supra* note 52, at 48.

98 Arielle R. Baskin-Sommers & Karelle Fonteneau, *Correctional Change through Neuroscience*, 85 Fordham L. Rev. 423, 426 (2016).

99
*See generally* Justin D. Strong et al., *The Body in Isolation: The Physical Health Impacts of Incarceration in Solitary Confinement*, 15 PLoS ONE e0238510 (2020); Peter Scharff Smith, *The Effects of Solitary Confinement on Prison Inmates: A Brief History and Review of the Literature*, 34 Crime & J. 441 (2006).

